# Complete Chloroplast Genome of *Corethrodendron fruticosum* (Papilionoideae: Fabaceae): Comparative and Phylogenetic Analysis

**DOI:** 10.3390/genes14061289

**Published:** 2023-06-19

**Authors:** Tianxiu Niu, Chunyu Tian, Yanting Yang, Qian Liu, Lemeng Liu, Qibo Tao, Zhiyong Li, Zinian Wu

**Affiliations:** 1 Key Laboratory of National Forestry and Grassland Administration on Grassland Resources and Ecology in the Yellow River Delta, Qingdao Key Laboratory of Specialty Plant Germplasm Innovation and Utilization in Saline Soils of Coastal Beach, College of Grassland Science, Qingdao Agricultural University, Qingdao 266109, China; 17863909472@163.com (T.N.); taoqibo@qau.edu.cn (Q.T.); 2 Institute of Grassland Research, Chinese Academy of Agricultural Sciences, Hohhot 010010, China; tiancy2021@163.com (C.T.); yangyanting@caas.cn (Y.Y.); cheerliu1987@126.com (Q.L.); liulemeng@caas.cn (L.L.); lizhiyong01@caas.cn (Z.L.); 3 Key Laboratory of Grassland Resources and Utilization of Ministry of Agriculture, Hohhot 010010, China

**Keywords:** *Corethrodendron fruticosum*, chloroplast genome, codon usage, repeat analysis, phylogenetic relationship

## Abstract

*Corethrodendron fruticosum* is an endemic forage grasses in China with high ecological value. In this study, the complete chloroplast genome of *C. fruticosum* was sequenced using Illumina paired-end sequencing. The *C. fruticosum* chloroplast genome was 123,100 bp and comprised 105 genes, including 74 protein-coding genes, 4 rRNA-coding genes, and 27 tRNA-coding genes. The genome had a GC content of 34.53%, with 50 repetitive sequences and 63 simple repeat repetitive sequences that did not contain reverse repeats. The simple repeats included 45 single-nucleotide repeats, which accounted for the highest proportion and primarily comprised A/T repeats. A comparative analysis of *C. fruticosum, C. multijugum*, and four *Hedysarum* species revealed that the six genomes were highly conserved, with differentials primarily located in the conserved non-coding regions. Moreover, the *accD* and *clpP* genes in the coding regions exhibited high nucleotide variability. Accordingly, these genes may serve as molecular markers for the classification and phylogenetic analysis of *Corethrodendron* species. Phylogenetic analysis further revealed that *C. fruticosum* and *C. multijugum* appeared in different clades than the four *Hedysarum* species. The newly sequenced chloroplast genome provides further insights into the phylogenetic position of *C. fruticosum*, which is useful for the classification and identification of *Corethrodendron.*

## 1. Introduction

*Corethrodendron fruticosum* (Leguminosae) is a subshrub distributed primarily in the grassland areas of eastern Inner Mongolia and western northeast China [1]. It is suitable for planting on semi-fixed or flowing sand with good aeration and water [2]. *C. fruticosum* is a valuable forage grass that is unique to China that can also be employed for windbreak and sand fixation [3]. Moreover, it is resistant to drought, high temperatures, and wind erosion, with a low seed germination rate, strong asexual reproduction, and vigorous growth in the third year of planting [2]. Currently, *C. fruticosum* is broadly distributed in arid and semi-arid areas in northern China as an excellent forage grass and for wind and sand control [4]. In particular, mild saline stress stimulates *C. fruticosum* root growth at the end of the growth period [5]. Meanwhile, light sand burial accelerates the growth of *C. fruticosum* meristems, whereas high sand burial (at depths of 80–100% of the *C. fruticosum* plant height) weakens the survival and growth of *C. fruticosum* meristems [6]. *C. fruticosum* was originally classified as *Hedysarum* [7]; however, more recent morphological and molecular phylogenetic evidence supports that *Corethrodendron* is a species that is independent of *Hedysarum* [8,9]. The primary *Corethrodendron* species include *C. scoparium*, *C. multijugum,* and *C. fruticosum.*

Chloroplasts are plastids that are common in land plants, algae, and protists that function as semi-autonomous organelles possessing the chloroplast genome or plastome as their genetic material [10]. With the development of next-generation sequencing (NGS) technologies, chloroplast genome sequencing has becoming a research hot spot. Chloroplast genomes have crucial roles in phylogeny, species identification, and crop breeding [11]. The chloroplast genomes of most plants comprise four regions: a large single-copy region, a small single-copy region, and two inverted repeat regions (IR) that act as chloroplast spacers between the large and small single-copy regions. There are four regions: a large single-copy region, a small single-copy region, and two inverted repeat regions (IR) that act as spacers between the large and small single-copy regions in the chloroplast genomes of most species [12,13,14]. Chloroplast genomes are typically 115–160 kb in length and generally encode 110–130 genes [15]. The variation in genome size is primarily influenced by variation in the IR region length [16,17,18]. However, certain legume species, including *Medicago truncatula* [19], *Pisum sativum* [20], and *Caragana microphylla* [21], exhibit a loss of the IR region and are collectively designated as the IR-lacking clade (IRLC) [22,23]. In other species, such as *Pinus thunbergia* [24], the IR region undergoes contraction, while in others, such as *Pelargonium hortorum* [25], it is expanded.

The chloroplasts of *Nicotiana tabacum* [26] and *Marchantia polymorpha* [27] have been successively sequenced and annotated, and the number of sequences has rapidly increased. In fact, 11,946 chloroplast genomes from 19,388 species, including 604 Leguminosae, have been integrated and curated in the chloroplast genome information resource (CGIR, https://ngdc.cncb.ac.cn/cgir accessed on 25 May 2023) [28,29]. In particular, the chloroplast genomes of *C. multijugum* and four *Hedysarum* species (*Hedysarum semenovii*, *Hedysarum polybotrys*, *Hedysarum petrovii*, and *Hedysarum taipeicum*) have been sequenced and annotated, thereby revealing that all five species have lost the IR regions [30,31,32].

In this study, the complete chloroplast genome of *C. fruticosum* was sequenced and annotated; it was then compared to those of *C. multijugum* and the four *Hedysarum* species chloroplast genome sequences mentioned above. Repeat sequences, simple sequence repeats (SSRs), nucleotide diversity (Pi), and the evolution of the six species were comparatively studied to gain further insight into the chloroplast genome of *C. fruticosum*. Additionally, we created a phylogenetic tree based on the 30 species chloroplast genome sequences in order to study their evolutionary relationships.

## 2. Materials and Methods

### 2.1. DNA Extraction and Sequencing, Genome Assembly, and Annotation

One *C. fruticosum* was collected from Ordos, Inner Mongolia, China (40.42° N, 110.04° E) and stored at the National Medium Term Genebank Forage Germplasm (Hohhot, China). Total genomic DNA was extracted from fresh leaves using a TIANamp Genomic DNA Kit (Tiangen Biotech Co., Ltd., Beijing, China). NGS was performed using a MiSeq PE150 platform to generate 150 bp-paired reads. The chloroplast genome was de novo assembled using GetOrganelle [33] and annotated using the Plastid Genome Annotator tool [34]. Geneious V9.0.2 was used to manually fix incorrect annotations of initiation codons and to stop codons made by Plastid Genome Annotator [35,36]. The chloroplast genome sequences of *C. multijugum* (NC_069301.1), *H. taipeicum* (NC_046493.1), *H. polybotrys* (MZ322397.1), *H. semenovii* (NC_047344.1), and *H. petrovii* (MT120797.1) were obtained from GenBank.

### 2.2. Identification of Repetitive Sequences and SSRs

Repetitive sequences, including forward repeats, reverse repeats, complementary repeats, and palindromic repeats, were identified using REPuter [37] under the following minimal repeat lengths: 30, with a hamming distance of 3. SSRs were identified using MISA [38], with the following parameter settings: unit size (nucleotide) minimum repeat: 1–10, 2–6, 3–4, 4–-3, 5–3, and 6–3. The minimum distance between two SSRs of 100 bp.

### 2.3. Analysis and Comparison of Genome Structures

Relative synonymous codon usage (RSCU) was calculated for all codons using CodonW v.1.4.2 (https://codonw.sourceforge.net accessed on 16 April 2023). The number of codons of protein-coding genes in the *C. fruticosum* chloroplast genome was determined. Pi values and sequence polymorphisms of *C. fruticosum*, *C. multijugum*, and the four *Hedysarum* species were analyzed using DNAsp v.6.10 [39]. The complete chloroplast genome sequences of *C. fruticosum, C. multijugum, H. taipeicum*, *H. polybotrys*, and *H. semenovii* were compared using mVISTA, with *H. petrovii* as the reference sequence [40] using default parameters.

### 2.4. Phylogenetic Analysis

The chloroplast genome sequences of *C. fruticosum* and 30 other species retrieved from NCBI were used to construct a phylogenetic tree using *Arabidopsis thaliana* and *Oryza sativa* as outgroups (Table S1). The concatenated protein-coding genes were used for phylogenetic analysis. All sequences were aligned using MAFFT (parameter default) [41]. Trees were constructed using the maximum likelihood and Bayesian methods. The best-fitting substitution model was selected using Modeltest 3.7 [42]. The maximum likelihood tree was constructed using IQ-TREE 1.6.12 [43] with the GTR + F + R4 model, and branch support was analyzed using bootstrap analysis with 1000 replicates. The Bayesian tree was constructed using MrBayes v.3.2.6 [44] with the GTR +F + I + G4 model.

## 3. Results

### 3.1. Genomic Characteristics of the C. fruticosum Chloroplast Genome

**Figure 1:**
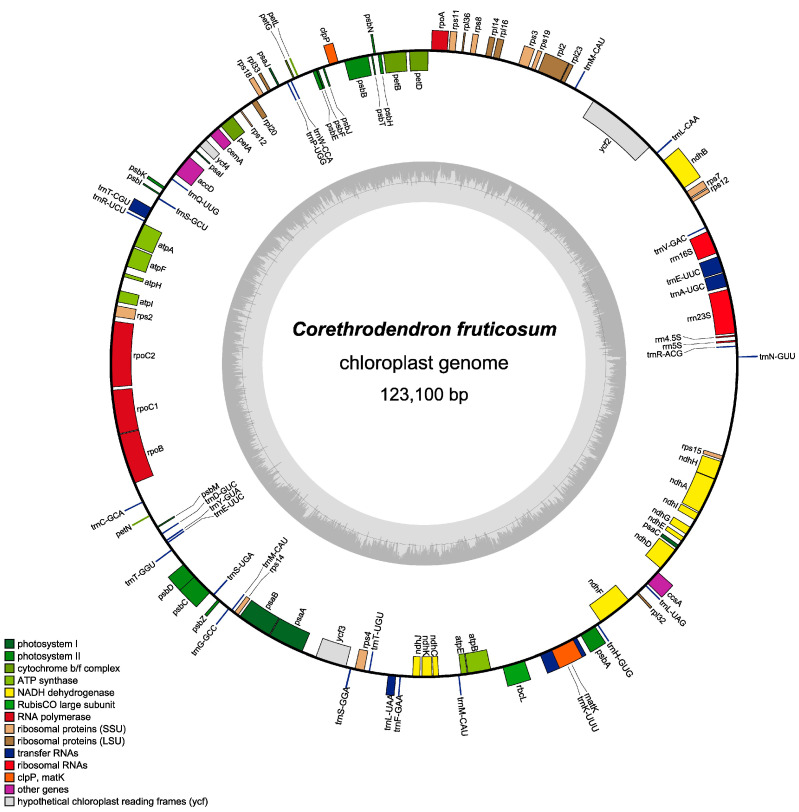
Gene map of the *C. fruticosum* chloroplast genome. Genes distributed outside the outer circle are transcribed in the clockwise direction; those distributed inside the outer circle are transcribed in the counterclockwise direction. The dashed darker gray area of the inner circle indicates GC base content; the lighter gray indicates AT base content. Different functional groups genes are coded with different colors.

We sequenced and annotated the *C. fruticosum* chloroplast genome (Figure 1), which was missing a copy of the IR region; this supports the placement of *C. fruticosum* in the IRLC in Papilionoideae. The chloroplast genome of *C. fruticosum* was calculated as 123,100 bp that comprised 105 genes (Table 1), including 74 protein-coding genes, 4 rRNA-coding genes, and 27 tRNA-coding genes. Forty-three genes were associated with photosynthesis. The genes associated with transcription included 8 encoding ribosomal large subunits, 11 encoding ribosomal small subunits, and 4 encoding DNA-dependent RNA polymerases.

Genes associated with transcription were also identified, and they comprised four genes encoding ribosomal RNAs: *rrn16S*, *rrn23S*, *rrn4.5S*, and *rrn5S*. What is more, six other genes and two unknown genes, *ycf3*, and *ycf4*, were identified. The gene *ycf2* was also catalogued to an unknown gene before, but it was identified as a component of the translocon recently [45]. Of the 15 genes in the introns, all except *ycf3* contained one intron, while *ycf3* contained two. Additionally, *trnK-UUU* contained the longest amount of introns (2451 bp) and was discovered to be the longest intron (Table 2).

### 3.2. Codon Usage Analysis of Protein-Coding Genes

**Figure 2:**
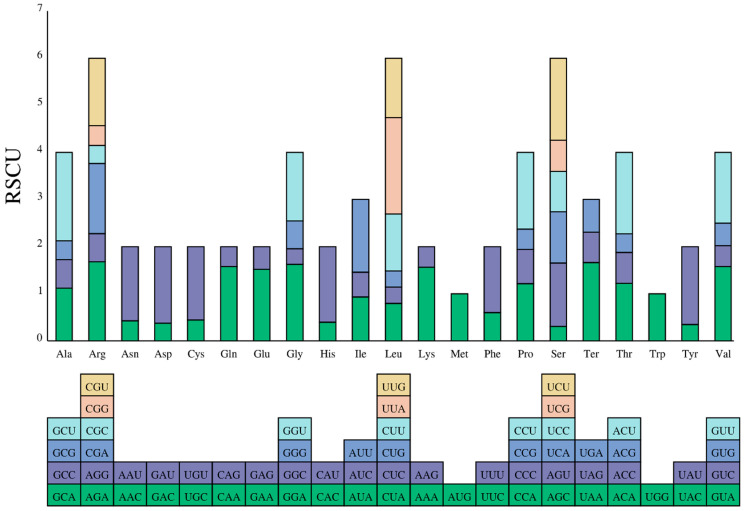
Codon usage (RSCU) in protein-coding genes in the *C. fruticosum* chloroplast genome.

The chloroplast genome of *C. fruticosum* was found to contain 19,798 codons. Arg was the most common amino acid, whereas Trpwas the least common (Table 3). Even if the termination codon is counted in, the most common codon was ATT, which appeared 911 times and encoded Ile, whereas the least common codon was TGA encoding Ter, which appeared only 17 times. The RSCU reflects the ratio of the actual codon usage frequency compared to the expected frequency (Figure 2) [46]. In the *C. fruticosum* chloroplast genome, most codons with a high RSCU end in A/T bases, while the codon TTG occurred in Leu (RSCU > 1). Met and Trp were encoded by only one codon and had no codon preference.

### 3.3. Repeat Analysis

**Figure 3:**
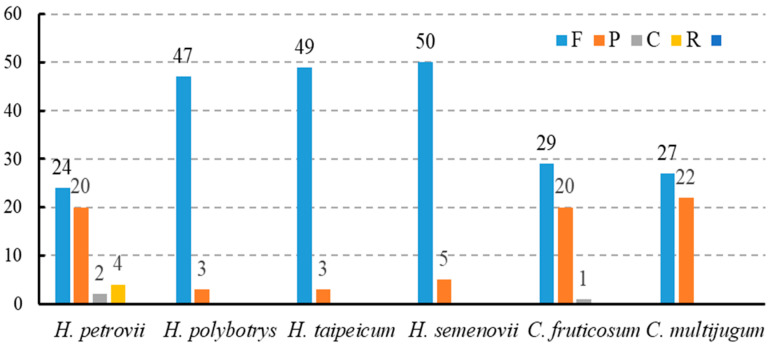
Numbers of repetitive sequences in the complete chloroplast genomes of *C. fruticosum*, *C. multijugum*, and four *Hedysarum* species.

In the *C. fruticosum* chloroplast genome, 50 repetitive sequences (Figure 3) were identified, including forward, complementary, and palindromic repeats. No reverse repeat sequences were detected. Forward repeats (58%) accounted for the largest proportion of repetitive sequences, followed by palindromic (40%) and complementary (2%) repeats. Meanwhile, the *C. multijugum* chloroplast genome lacked complementary repeats; however, it contained similar numbers of forward and palindromic repeats as *C. fruticosum*. The chloroplast genomes of *H. polybotrys*, *H. taipeicum,* and *H. semenovii* only contained forward and palindromic repeats, with the former found to be the most abundant, accounting for 94%, 94.2%, and 90.9%, of the repetitive sequences, respectively. In contrast, four types of repetitive sequences were identified in the chloroplast genome of *H. petrovii*: forward (48%), reverse (8%), complementary (4%), and palindromic (40%) sequences. The genes containing the most repetitive sequences in *C. fruticosum* were *rps15* and *trnN-GUU*, which contained palindromic (2) and forward (12) sequences (Table S3).

**Figure 4:**
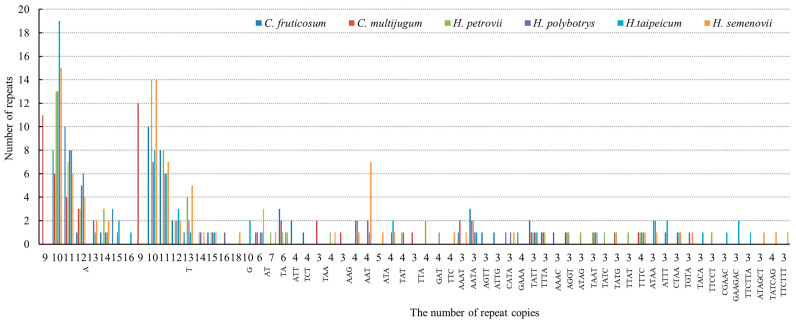
Numbers of SSRs of *C. fruticosum*, *C. multijugum*, and four *Hedysarum* species.

Sixty-three SSRs (Table 4) were ascertained in the chloroplast genome of *C. fruticosum*, with single-nucleotide repeats comprising 10–15 of repeat units, dinucleotide repeats comprising 6 repeat units, trinucleotide repeats comprising 4 repeat units, and tetranucleotide repeats comprising 3 repeat units (Figure 4). There were markedly more mononucleotide repeats in these six species than in the compound SSRs (Table S2). Of the six Fabaceae species, *C. multijugum* had the fewest compound SSRs (7), while *H. taipeicum* and *H. semenovii* had the most (16). Mononucleotide repeats included only the A/T in *C. fruticosum*, and *C. multijugum*, while the G/C in *H. taipeicum*, *C. fruticosum* and *H. polybotrys* had a single-nucleotide repeat. *C. fruticosum*, *C. multijugum*, and *H. polybotrys* had no pentanucleotide or hexanucleotide repeats, whereas *H. petrovii* had one hexanucleotide repeat (AAAGG/CCTTT). Regarding *C. fruticosum*, mononucleotide repeats (45) were the most abundant, followed by tetranucleotide repeats (12). *H. petrovii* and *H. taipeicum* each carried one pentanucleotide repeat, whereas *H. taipeicum* and *H. semenovii* had two and three hexanucleotide repeats, respectively. The chloroplast genome of *H. taipeicum* had one type of tetranucleotide repeat (AATC/ATTG) that was not found in the other *Hedysarum* species. The number and variety of SSRs in the *rps15* and *trnN-GUU* was also the highest in *C. fruticosum* (Table S4).

### 3.4. Comparative Analysis of the C. fruticosum Chloroplast Genome

**Figure 5:**
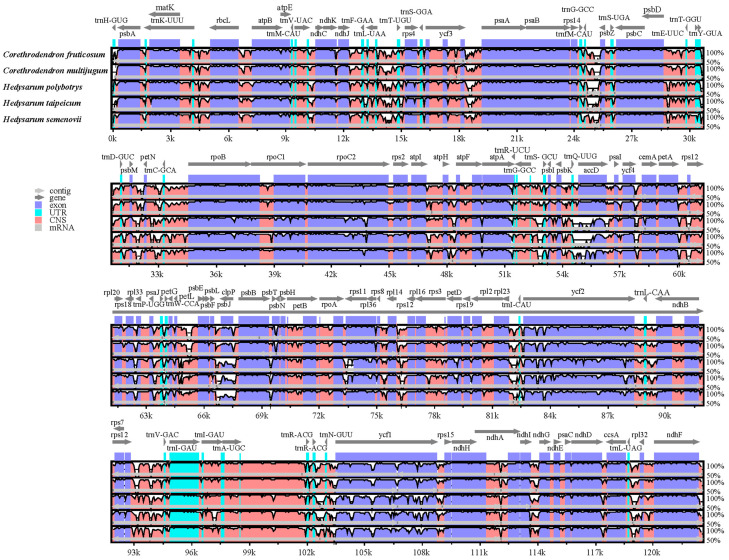
Global alignment of six chloroplast genomes using the *H. petrovii* genome as a reference sequence. The horizontal axis indicates the coordinates in the chloroplast genome. The vertical scale represents the average percentage of sequence similarity in the aligned regions, which ranged between 50% and 100%.

A comparison of the overall sequence variation in the chloroplast genomes using mVISTA revealed that the six chloroplast genomes were highly conserved (Figure 5). The gene intergenic regions of *ycf3-psaA*, *trnG-GCC-psbZ*, *trnT-GGU-psbD*, *ndhC-trnV-UAC*, *psbE-petL*, *rpl16-rpl14*, *trnI-CAU-rpl123*, *trnR-ACC-trnN-GUU*, *rps12-trnV-GAC*, *ndhI-ndhG*, *ndhF-rpl32*, and *rpl32-trnL-UAG* exhibited high variation and were located in the conserved non-coding regions (CNS) of the chloroplast genomes of these six species. In the exonic region, *rpoB*, *rpoC2*, *ycf1*, *ycf2*, and *clpP* exhibited significant differences. However, in all the studied chloroplast genomes, the regions with evident differences were primarily observed in the CNS.

**Figure 6:**
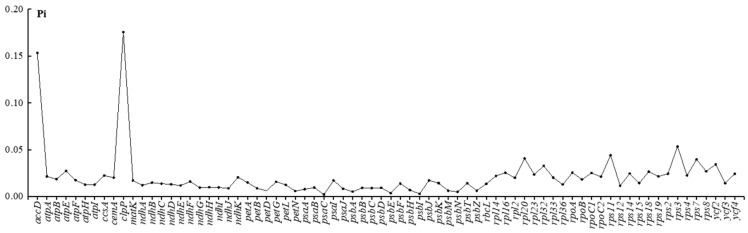
Line graph showing the gene nucleotide diversity (Pi) value of six Fabaceae species. X-axis: gene names. Y-axis: Pi value.

We calculated the Pi values of the six Fabaceae species to further clarify the variation in the coding regions (Figure 6). Although most sequences were relatively conserved (Pi < 0.01), *accD* and *clpP—*encoding an acetyl-CoA carboxylase subunit and protease, respectively—had high Pi values. Moreover, we identified four hotspot regions with Pi > 0.04 (*rps3*, *rps11*, *rps7*, and *rpl20*), all of which were related to ribosome subunit formation during transcription in plants.

### 3.5. Phylogenetic Analysis

**Figure 7:**
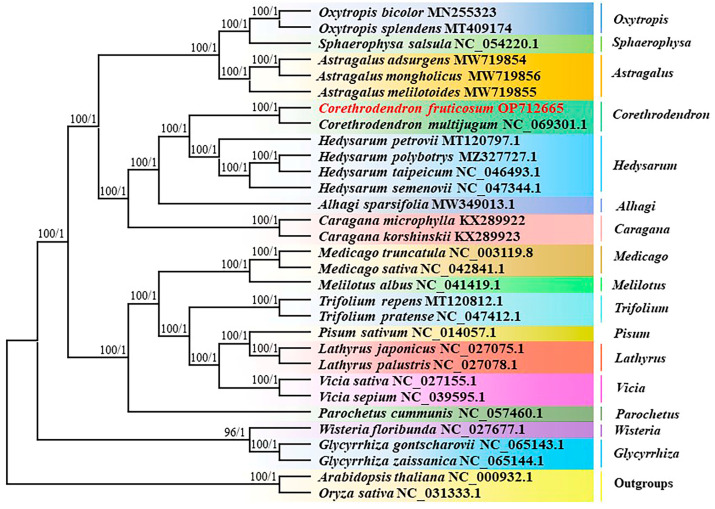
Phylogenetic tree of *C. fruticosum* and 30 other representative Fabaceae species based on 69 concatenated protein-coding genes. *Arabidopsis thaliana* and *Oryza sativa* were used as outgroups. Numbers associated with branches represent maximum likelihood values/Bayesian inference values (posterior probability).

The topology of the phylogenetic tree comprising 16 genera and 31 species of Papilionoideae, and the taxonomic agreement of *O. sativa* and *A. thaliana* as outgroups of Papilionoideae, had strong bootstrap support (Figure 7). *Corethrodendron* was independent of *Hedysarum*, whereas *C. fruticosum* and *C. multijugum* formed a high-support branch with the four *Hedysarum* species. However, among *Hedysarum* species, the closest relatives were *H. polybotrys* and *H. taipeicum*, which formed a branch with *H. semenovii* with high support. Moreover, besides *Hedysarum* species, *Corethrodendron* was more closely related to *Alhagi* and *Caragana* compared with the other Papilionoideae genura.

## 4. Discussion

### 4.1. Sequence Variation in C. fruticosum

In the present research, five previously published complete chloroplast genomes were compared to that of *C. fruticosum*. No significant structural rearrangements were found in the genome of *C. fruticosum*, except for the deletion of the IR region. The gene contents and sequences of *Corethrodendron* and *Hedysarum* were highly conserved. We found that the codons in the *C. fruticosum* chloroplast genome exhibited a preference for A/T bases, which is often found in higher plants [47,48,49]. Accordingly, the GC content in *C. fruticosum*, as with *C. multijugum* and four *Hedysarum* species, was low [30,31,32].

The results of mVISTA analysis indicated that the length and gene order of the chloroplast genomes in these six plant were highly uniform; however, the CNS exhibited greater variation than other regions. As is consistent with previous studies, certain gene intergenic regions can be used as DNA barcodes for plant classification and identification [50,51]. More specifically, the divergent CNS regions in *C. fruticosum*, namely, *psbE-petL* and *ndhF-rpl32*, might prove effective when developed as DNA barcodes. Similarly, the chloroplast *matK*, *trnL-trnF*, and *psbA-trnH* sequences can be used as a basis for *Hedysarum* taxon delimitation [8,9]. The two genes with the highest Pi values in the coding region in the *C. fruticosum* chloroplast genome were *accD* and *clpP*. *ClpP* encodes a protease that is involved in chloroplast protein homeostasis and gene expression regulation [52]. *AccD* encodes the β-carboxyltransferase subunit of acetyl-CoA carboxylase [53]. Acetyl-CoA carboxylase is the rate-limiting enzyme in fatty acid biosynthesis, and its expression is induced by light [54,55,56]. Hence, these two genes may be responsible for the superior ability of *C. fruticosum* to grow on sand compared with the other four *Hedysarum* species [57]. As such, they have potential applications in *C. fruticosum* related to high light efficiency and stress tolerance breeding.

### 4.2. Repeat Sequences

The main source of duplication, rearrangement, and deletion events occurring in the chloroplast genome are repetitive sequences [58]. *C. fruticosum* had a low variety and few repetitive sequences. *H. petrovii* was the most similar to *C. fruticosum* in terms of the types and numbers of repetitive sequences, which may reflect the degree of relatedness between these species. Chloroplast SSRs are primarily located in non-coding regions and have the advantages of being highly conserved, endowing with uniparental inheritance, and having relative evolutionary independence [59,60,61]. Many plant species harbor chloroplast SSR markers [62]. The chloroplast SSRs in *C. fruticosum, C. multijugum,* and the four *Hedysarum* species primarily included poly-A/T and multi-base repeats, which is a consistent result with those found in other species in the IRLC clade [63]. Indeed, the chloroplast SSRs of *C. fruticosum, C. multijugum*, and the four *Hedysarum* species were highly variable, particularly for the composite SSRs. Hence, these SSRs can be used as molecular markers to differentiate *C. fruticosum* with other species and can provide a basis for studying the phylogeny and population of *C. fruticosum*.

### 4.3. Phylogeny of C. fruticosum

Our phylogenetic understanding of *Corethrodendron* is incomplete. *Corethrodendron* was originally classified as *Hedysarum* [7], and early phylogenetic studies of *Hedysarum* species used the sequence of the chloroplast gene *matK* for all Leguminosae [64,65]. Later, phylogenetic trees of *Hedysarum* were constructed using nuclear, gene intergenic regions and sequences of multiple chloroplast loci, including *matK*, *trnL-trnF*, and *psbA-trnH* [8,9]. During this period, a study used morphological data for the reclassification of *Hedysarum* [66]; the results were included in the Flora of China [1]. Collectively, these results highlight the independence of *Corethrodendron* from *Hedysarum*, which is supported by our findings. One study revealed that, among the species classified as Leguminosae IRLC, *Hedysarum* is more closely related to *Astragalus* than is *Medicago* [31], which is also supported by our findings. This may be due to the fact that the IR region of *Astragalus* is completely missing in *Hedysarum* and *Corethrodendron* [67], whereas the IR region in *Medicago* species, such as *Medicago truncatula*, is partially deleted [19]. The results of the present study indicate that *Corethrodendron* is more closely related to *Alhagi* and *Caragana* than to *Astragalus*. This may be due to most *Astragalus* species being herbaceous, whereas most others are subshrubs.

## 5. Conclusions

In this study, we sequenced and assembled the complete chloroplast genome of *C. fruticosum* and compared it to those of *C. multijugum* and four *Hedysarum* species, all of which belong to Papilionoideae. These species all belong to the IRLC. Their chloroplast genomes were found to be rich in repetitive sequences and SSRs, some of which can be used as molecular markers in genetic diversity analysis and *Corethrodendron* species identification. The marked differences in the CNS region can be used as novel DNA barcodes. The chloroplast genome of *C. fruticosum* had distinctly differentiated coding regions compared to the *Hedysarum* species. This further supports the independence of *Corethrodendron* from *Hedysarum*. However, the specific evolutionary relationship between *Corethrodendron* and *Hedysarum* remain unclear, and the few studies on other *Corethrodendron* are scarce. Collectively, this study provides useful information for the phylogenetic analysis and species identification of *Corethrodendron*.

## Figures and Tables

**Table 1:** Genes encoded by the chloroplast genome of *C. fruticosum*.

Gene Category	Gene Group	Gene Names
Photosynthesis	Subunits of Photosystem I	*psaA*, *psaB*, *psaC*, *psaI*, *psaJ*
	Subunits of Photosystem II	*psbA*, *psbB*, *psbC*, *psbD*, *psbE*, *psbF*, *psbH*, *psbI*, *psbJ*, *psbK*, *psbM*, *psbN*, *psbT*, *psbZ*
	NDH complex	ndhA *, ndhB *, *ndhC*, *ndhD*, *ndhE*, *ndhF*, *ndhG*, *ndhH*, *ndhI*, *ndhJ*, *ndhK*
	Subunits of cytochrome b/f complex	*petA*, petB *, petD *, *petG*, *petL*, *petN*
	Subunits of ATP synthase	*atpA*, *atpB*, *atpE*, atpF*, *atpH*, *atpI*
	Subunits of Rubisco	*rbcL*
Transcription	Large subunits of ribosomes	*rpl14*, *rpl16*, rpl2 *, *rpl20*, *rpl23*, *rpl32*, *rpl33*, *rpl36*
	Small subunits of ribosomes	*rps11*, rps12 *, *rps14*, *rps15*, *rps18*, *rps19*, *rps2*, *rps3*, *rps4*, *rps7*, *rps8*
	DNA-dependent RNA polymerase	*rpoA*, *rpoB*, rpoC1 *, *rpoC2*
	rRNA genes	*rrn16S*, *rrn23S*, *rrn4.5S*, *rrn5S*
	tRNA genes	trnA-UGC *, *trnC-GCA*, *trnD-GUC*, *trnE-UUC*, trnE-UUC *, *trnF-GAA*, *trnG-GCC*, *trnH-GUG*, trnK-UUU *, *trnL-CAA*, trnL-UAA *, *trnL-UAG*, *trnM-CAU(3)*, *trnN-GUU*, *trnP-UGG*, *trnQ-UUG*, *trnR-ACG*, *trnR-UCU*, *trnS-GCU*, *trnS-GGA*, *trnS-UGA*, trnT-CGU *, *trnT-GGU*, *trnT-UGU*, *trnV-GAC*, *trnW-CCA*, *trnY-GUA*
Other genes	C-type cytochrome synthesis genes	*ccsA*
	Envelope membrane proteins	*cemA*
	Proteases	*clpP*
	Subunits of acetyl-CoA carboxylase	*accD*
	Maturases	*matK*
	Components of the translocon	ycf2 *
Unknown	Conserved open reading frames	ycf3 **, *ycf4*

* Intron number; Gene (3): number of copies of multiple gene copies; ** meant *ycf3* owned two introns.

**Table 2:** Size of introns and exons in split genes in the *C. fruticosum* complete chloroplast genome.

Gene	Strand	Start	End	Exon I (bp)	Intron I (bp)	Exon II (bp)	Intron II (bp)	Exon III (bp)
*atpF*	−	54,104	55,196	172	685	407		
*ndhA*	−	113,391	115,680	553	1198	539		
*ndhB*	+	11,627	13,789	721	678	764		
*petD*	−	30,512	31,706	8	712	475		
*petB*	−	31,901	33,335	6	787	642		
*rpl2*	+	21,974	23,497	397	696	431		
*rpoC1*	−	63,215	66,006	430	737	1625		
*rps12*	−	10,591	43,117	114		258		
*ycf2*	−	14,983	19,560	2711	30	1837		
*ycf3*	−	85,395	87,263	124	714	236	787	132
*trnT-CGU*	+	51,384	52,138	35	677	43		
*trnL-UAA*	+	90,065	90,571	35	422	50		
*trnK-UUU*	+	99,750	102,272	37	2451	35		
*trnE-UUC*	−	5824	6855	32	960	40		
*trnA-UGC*	−	4918	5759	37	805	36		

**Table 3:** Codon counts in the *C. fruticosum* chloroplast genome.

Codon	Count	Codon	Count	Codon	Count	Codon	Count
TAA	41	GGC	119	ATG	470	AGT	325
TAG	16	GGG	212	AAC	192	TCA	263
TGA	17	GGT	525	AAT	702	TCC	207
GCA	332	CAC	94	CCA	252	TCG	160
GCC	179	CAT	378	CCC	151	TCT	420
GCG	117	ATA	549	CCG	90	ACA	313
GCT	555	ATC	312	CCT	338	ACC	168
TGC	52	ATT	911	CAA	558	ACG	100
TGT	182	AAA	762	CAG	150	ACT	443
GAC	140	AAG	212	AGA	308	GTA	443
GAT	607	CTA	281	AGG	111	GTC	124
GAA	755	CTC	122	CGA	273	GTG	134
GAG	237	CTG	121	CGC	71	GTT	422
TTC	339	CTT	426	CGG	77	TGG	342
TTT	794	TTA	722	CGT	263	TAC	124
GGA	587	TTG	446	AGC	74	TAT	588

**Table 4:** Number of SSRs of four *Hedysarum* species, *C. fruticosum*, and *C. multijugum*.

Species	Total SSRs	Compounds SSRs	Type					
			Mono-	Di-	Tri-	Tetra-	Penta-	Hexa-
***C. fruticosum***	63	8	45	3	3	12	0	0
***C. multijugum***	59	7	43	3	4	9	0	0
***H. semenovii***	68	10	50	1	6	11	0	0
***H. taipeicum***	80	16	59	2	4	11	1	3
***H. semenovii***	88	16	60	5	12	8	0	3
***H. petrovii***	76	14	56	1	6	12	1	0

## Data Availability

The complete chloroplast genome sequences of the *C. fruticosum* we sequenced were deposited in the NCBI; the GenBank accession number is the following: OP712665.

## Supplementary Materials

The following supporting information can be downloaded at: https://www.mdpi.com/article/10.3390/genes14061289/s1, Table S1: GenBank accession numbers of chloroplast genomes; Table S2: SSR motif numbers in the *C. fruticosum*, *C. multijugum*, and four *Hedysarum* species chloroplast genomes; Table S3: Repeat sequences in the Corethrodendron fruticosum chloroplast genome; Table S4: SSR locations in the Corethrodendron fruticosum chloroplast genomes.
